# Case of Hydrophobia in South Easton

**Published:** 1851-04

**Authors:** Abraham Stout

**Affiliations:** Easton, Pennsylvania


*Case of Hydrophobia in South Easton. Communicated by* Abraham Stout, M. D., of Easton, Pennsylvania.

Mr. Bartholomew, of South Easton, owned a small dog, which he presented, about the beginning of March, 1848, to Mr. Rose, of our borough. After the dog had remained at Mr. Rose's, during the night, he returned to his old home. In the afternoon of the same day on which the dog returned, he bit the daughter of Mr. Jacob Brown in the face. The dog then disappeared and has not been heard of since. The wounds in the little girl's face were healed, and she enjoyed very good health till Thursday the 20th of April, when Dr. Slough was called to prescribe for irritability of the stomach, attended by occasional vomiting. On Friday she appeared better, but seemed unusually depressed in spirits. At ten o'clock, P. M., on Saturday, the 22d, her father offered her a drink of coffee, and the moment she saw it, she started back in apparently frightful agony.

The father admonished her and told her not to do so; she replied that she could not help it. She then took coffee into her mouth but swallowed it with great difficulty. The sight of coffee or water continued more and more dreadful to her till Sunday morning at six o'clock, when Dr. Slough was again called in. The Doctor was at once satisfied that the girl, about five years old, labored under hydrophobia, and convinced the family of the fact by pouring water, in her presence, from one vessel into another, which threw her into painful spasms. Pressure on the cicatrix of the wounds had the same effect. Dr. Slough proposed a consultation with me. We met at ten o'clock A. M., on Sunday the twenty-third, and concluded to give chloroform and ether a trial in the case. As neither Dr. Slough or myself had used these powerful agents, we requested Mr. Crane, a practical dentist, to put the patient under their influence. Mr. Crane used the chloroform in the first place, and soon put the patient under its influence, when all the symptoms of hydrophobia disappeared; but the disease returned, with an apparent increase of violence, and continued so until the chloroform was again resorted to. After several repetitions of the chloroform, without gaining any advantage over the disease, ether was resorted to; but like the chloroform, it had no farther effect than to suspend the disease so long as the system was kept under its influence, and when consciousness returned, the symptoms of hydrophobia increased, and the sight of a polished surface, such as Mr. Crane's inhaler, would cause her to throw herself back, and bring on spasms of the muscles. No other medicine was used, either general or local, nor was she bled. She expired at five o'clock P. M., six hours after the first use of the chloroform, in the presence of

Doctors Swift, Slough, Sloan, Abernethy, Wagner, Innis, and myself.

Sixteen hours after the patient's decease, I inspected the body, in the presence of Doctors Swift, Slough, Wagner, and Abernethy. The upper part of the scull was first removed, when the blood vessels of the dura mater were seen to be unusually distended, and bled so much from their ruptured orifices as to mark the surface of the dura mater with numerous streaks. After removing the dura mater, the smell of sulphuric ether was as apparent from the brain, as it is from the mouth of a bottle that contains some of that fluid. I called the attention of the physicians who were present to it, and they all acknowledged the truth of the fact. The brain was greatly engorged, and in a groove formed by two of the convolutions, a coagulum of blood existed. By slicing off portions of the medullary part of the brain, the blood vessels, on the surfaces, formed unusually large red spots. No extravasated blood existed in the cavities of the brain.

The lungs appeared in a perfectly normal state. No engorgement of the lungs existed, and from no part of them could we perceive the smell of sulphuric ether; nor did we discover any traces of ether in the cavity of the abdomen or in the stomach and bowels.

*Remarks.*—From a superficial glance at the above case, it does not appear to possess sufficient interest to deserve a place in a medical journal, nor to be worthy of the time required for its perusal by the respective readers, inasmuch as the symptoms of the disease and the termination of the case are like all the numerous cases of hydrophobia that are upon record; but when we take into consideration the discovery of ether, in its pure and undecomposed state, in the brain, and the only manner in which it could possibly have found its way there, it will incontrovertibly prove the manner in which aerial poisons find their way into our systems, to act as remote causes of fevers and perhaps other diseases. And, moreover, the fact of extensive engorgement of the brain, produced, without doubt, by the chloroform and ether, points out the organ upon which the inhaled poisons principally act.

As far as my experience has gone in the use of chloroform and ether, I feel satisfied that they do not operate upon any disease directly, but upon life itself. A disease is only brought to a stand by them in proportion and as long as they suspend life. When their effects cease the disease will resume its former progress. As long as we are not in possession of a gage (Vitalometer) to determine the degree of life that we can suspend with safety, so long I shall consider chloroform and ether dangerous agents; and if they are continued to to be used at random, they will in the future, as they have hitherto done in many instances, supersede the disease in destroying life.

